# ﻿Structure of the genetic variation in the common springtail *Isotomiellaminor* (Hexapoda, Collembola) from contrasting habitats: evidence for different genetic lineages at a regional scale?

**DOI:** 10.3897/zookeys.1245.152112

**Published:** 2025-07-14

**Authors:** Mária Fedičová, Natália Raschmanová, Martina Žurovcová, Vladimír Šustr, Ľubomír Kováč

**Affiliations:** 1 Department of Zoology, Institute of Biology and Ecology, Faculty of Science, Pavol Jozef Šafárik University, Šrobárova 2, SK-04154 Košice, Slovakia; 2 Institute of Entomology, Biology Centre AS CR v. v. i., Branišovská 31, CZ-37005 České Budějovice, Czech Republic; 3 Institute of Soil Biology and Biogeochemistry, Biology Centre AS CR v. v. i., Na Sádkách 702/7, CZ-37005 České Budějovice, Czech Republic

**Keywords:** Environmental conditions, evolution relationships, genetic variability, soil fauna, springtail populations, ubiquitous species

## Abstract

Although *Isotomiellaminor* (Schäffer, 1896) (Collembola) is widely distributed in temperate regions, it is one of the less-studied species genetically. The genetic variability and its structure in the common springtail *I.minor* were investigated on a regional geographic scale using mitochondrial (COI) and nuclear (28S rDNA) markers. A total of nine populations from urban habitats of the Košice city agglomeration and four populations from natural sites of the karst landscape were used for the present study carried out in the Western Carpathians, Slovakia. Up to nine cryptic lineages (MOTUs - molecular operational taxonomic units) were independently recognised by two molecular delimitation methods. In addition, high genetic distances between lineages were observed (p-dist: 10.87−22.75% and K2p: 11.98−27.22%), comparable to the genetic distances between species. This study showed that urban and natural habitats harbour significantly different genetic lineages. Limited dispersal of MOTUs (lineages) between natural and urban populations was also supported by analysis of molecular variance (AMOVA). While the *I.minor* populations at urban sites were mixtures of different lineages, the populations at natural sites were monophyletic and their haplotypes/genetic lineages were clearly grouped by individual sites. Possible ecological filtering between urban and natural environments within MOTUs is discussed with respect to the evolution of parthenogenetic species *I.minor* in this habitat complex.

## ﻿Introduction

Springtails (Collembola) inhabit soils of all continents from the Arctic to the Antarctic, with high species diversity in Europe (Potapov et al. 2023). Although about 9,500 species have been described to date (Bellinger et al. 1996–2024), the diversity of this group is highly underestimated. Cicconardi et al. (2013) estimated overall number of 50,000 Collembola species, however, the global species richness could be at least an order of magnitude greater than this estimate. Undoubtedly, this is due to a lack of experts and slow progress in species descriptions, and the widespread occurrence of cryptic species. Cryptic species sensu lato are defined as genetically distinct species that are difficult to identify visually but have some unique phenotypic characters (Chenuil et al. 2019). Cryptic diversity, i.e., the presence of cryptic species in the studied taxon (Shin and Allmon 2023), is considered a common phenomenon in Collembola and has been observed in many genera, such as *Entomobrya* (Katz et al. 2015), *Heteromurus* (Lukić et al. 2019), *Lepidocyrtus* (Mateos 2008; Zhang et al. 2018b), *Parisotoma* (Porco et al. 2012; von Saltzwedel et al. 2017; Striuchkova et al. 2024), *Pogonognathellus* (Felderhoff et al. 2010), *Pseudosinella* (Kováč et al. 2023), *Pygmarrhopalites* (Katz et al. 2018), *Tomocerus* (Zhang et al. 2014), and others.

The widespread springtail *Isotomiellaminor* (fam. Isotomidae) is an edaphic, parthenogenetic, and ubiquitous species with a broad Holarctic distribution beyond the high Arctic (Potapov 2001). In the temperate zone it is common across the elevational gradient from lowlands to subalpine forests. The species is especially numerous in mountain spruce and beech forests, but less abundant in thermophilous environments, such as grasslands, meadows, and urban areas (Fountain and Hopkin 2004; Kuznetsova 2006; Fiera 2009; Salmon et al. 2014; Heiniger et al. 2015; Santorufo et al. 2014, 2015; Raschmanová et al. 2016). Although this species is morphologically and ecologically well characterised, there is only one study on its genetic variability, in which several genetic lineages were described from different regions of Europe (von Saltzwedel et al. 2016).

Recent studies from anthropogenically contrasting environments by Zhang et al. (2018b), Striuchkova (2023), and Striuchkova et al. (2022, 2024) have documented distinct genetic lineages of morphologically homogenous and widespread species, such as *Lepidocyrtuslanuginosus* (Gmelin, 1788) and *Parisotomanotabilis* (Schäffer, 1896). These authors suggested that the cryptic species in *L.lanuginosus* were sorted by habitat type. In the parthenogenetic *P.notabilis*, the most diverse genetic composition of populations was found in forest habitats, whereas the least diverse was at urban sites with a high anthropogenic influence. The authors stated that the distribution of genetic lineages may be associated with the degree of habitat disturbance. Similarly, von Saltzwedel et al. (2014) supposed that environmental conditions of habitats may select for different lineages, respectively suggested establishment of lineages due to environmental filtering. In this study focused on contrasting environments, forests and grasslands were inhabited by different genetic lineages of the parthenogenetic mite *Oppiellanova* (Oudemans, 1902). The divergence of lineages by habitat may be preliminarily used in studies; however, there is an important challenge for studies integrating molecular and physiological data to assess whether molecularly defined lineages from contrasting habitats exhibit specifical ecophysiological traits. It is also important to note that the relationships between genetic lineages and ecophysiological traits have not yet been sufficiently investigated in Collembola. Screening of mitochondrial or nuclear genetic markers as well as investigation of transcriptomics especially in natural (non-laboratory) populations could shed more light on this relationship (Thorne et al. 2011; Beet et al. 2022).

In the present study we focused on genetic variability of *I.minor* at two contrasting ecosystems: a city agglomeration as an urban environment and forested karst habitats as a natural environment. Based on the recent studies that revealed distinct genetic lineages of morphologically homogeneous, widespread collembolan species (Porco et al. 2014; von Saltzwedel et al. 2016; Zhang et al. 2018b; Striuchkova et al. 2024), we hypothesised that this parthenogenetic isotomid from the habitat complex might represent different genetic lineages that could be more frequent in contrasting environments. The aim of the present study was to examine the structure of genetic variability of *Isotomiellaminor* in contrasting habitats at urban and natural environment using a combined analysis of mitochondrial and nuclear markers.

## ﻿Materials and methods

### ﻿Sampling and site characteristics

**Figure 1.**
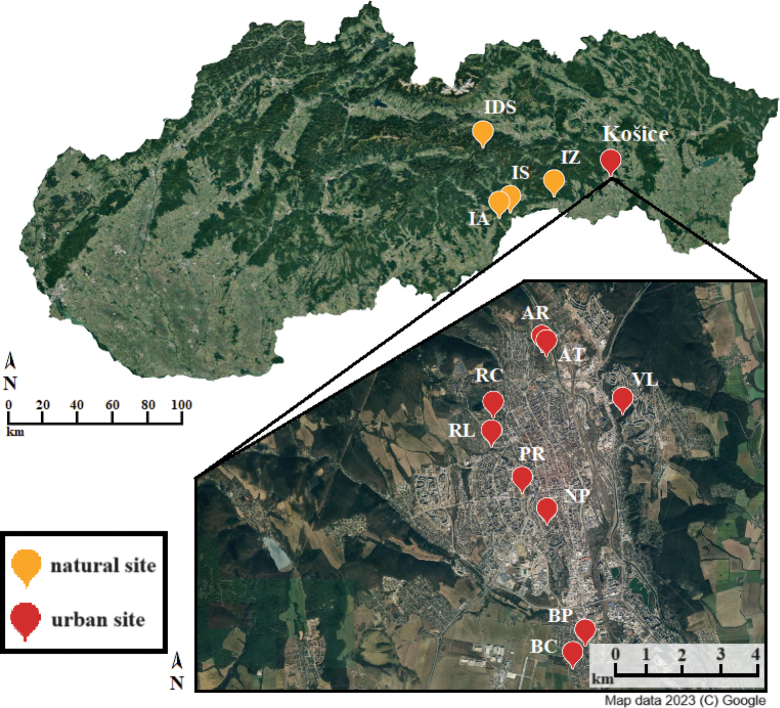
Location of the urban and natural sites of *Isotomiellaminor* populations in Slovakia. For abbreviations of sampling sites, see the Materials and methods section.

Thirteen populations of the species *Isotomiellaminor* were selected from two contrasting ecosystems composed of natural and urban habitats. Populations inhabiting habitats with a distinct level of anthropogenic effects served for this study, namely a ruderal habitat (AR, PR), a lawn (AT), a cemetery (BC, RC), a park (BP, NP), and a fragment of a woodland (RL, VL) in an urban agglomeration of Košice city, (~ 240 km^2^ and ~ 239,000 inhabitants), and populations from natural karst sites located near cave entrances and at a gorge bottom in the Slovak Karst (IS, IA, IZ) and Slovak Paradise (IDS) (Western Carpathians, Slovakia; Table 1, Fig. 1). The city of Košice is characterised by a mean annual air temperature +8.7 °C with annual average precipitation of 605 mm (Kaya et al. 2021). The Slovak Karst region is characterised by a mean annual air temperature ranging from +5.7 to +8.5 °C and annual average precipitation from 630 to 990 mm (Rozložník et al. 1994). In the Slovak Paradise National Park the mean annual air temperature ranges from +4.7 to +6.4 °C with annual average precipitation from 648 to 954 mm (Huňa et al. 1985). The annual temperature data at the urban and natural sites were analysed to document climatic differences. The mean soil temperature (T_mean_) was measured continually every 4 h (iButton DS1921G data-loggers) from May 2022 to May 2023 at each site (with exception of AT, NP, and PR sites, where no data is available) at a soil depth of 3 cm.

**Table 1.** Sampling design of *Isotomiellaminor* populations – sites, collection dates and number of specimens analysed. N – number of specimens. For abbreviations of sampling sites, see the Materials and methods section.

Sampling sites	N	Date
**Urban**		
AR – Ruderal habitat, Anička (Košice)	2	21 Oct 2021
AT – Lawn, Anička (Košice)	7	21 Oct 2021
BC – Cemetery (Košice - Barca)	7	21 Oct 2021
BP – Park (Košice - Barca)	5	21 Oct 2021
NP – Park (Košice)	4	21 Oct 2021
PR – Ruderal habitat, Starý pivovar (Košice)	6	21 Oct 2021
RC – Cemetery, Rozália (Košice)	4	21 Oct 2021
RL – Fragment of the woodland, Račí potok (Košice)	4	21 Oct 2021
VL – Fragment of the woodland, Výmoľ (Košice)	11	21 Oct 2021
**Natural**		
IA – Thermophilous cornel-oak wood, at the entrance of Ardovská Cave	4	15 Nov 2017
IS – Thermophilous hornbeam, at the entrance of Silická ľadnica Ice Cave	4	4 Nov 2017
IZ – Fragment of maple–hornbeam wood, on the bottom of the Zádiel Gorge	3	15 Nov 2017
IDS – Coniferous wood, at the entrance of Dobšinská Ice Cave	9	4 Nov 2017

(**AR**) Ruderal habitat, Anička (Košice) – 48°44'52.617"N, 21°15'5.185"E, 210 m a.s.l., T_mean_: 10.3 ± 6.6 °C, site with dense grass cover and dominant herb *Solidagocanadensis*;

(**AT**) Lawn, Anička (Košice) – 48°44'48.762"N, 21°15'11.121"E 210 m a.s.l., site characterised by dense grass cover, maintained by regular mowing;

(**BC**) Cemetery (Košice-Barca) – 48°40'23.512"N, 21°15'44.923"E, 211 m a.s.l., T_mean_: 11.8 ± 8.5 °C, solitary trees *Tiliacordata* and *Acerplatanoides* with dense grass cover, site with intensive human disturbance;

(**BP**) Park (Košice-Barca) – 48°40'42.466"N, 21°16'1.115"E, 196 m a.s.l., T_mean_: 10.7 ± 6.9 °C, site with typical park trees *Tiliacordata* and *Acerplatanoides* and sparse herbal cover;

(**NP**) Park (Košice) – 48°42'25.737"N, 21°15'11.821"E, 203 m a.s.l., park trees with *Gledistschiatriacanthos* and *Acerplatanoides* and dense herbal cover;

(**PR**) Ruderal habitat, Starý pivovar (Košice) – 48°42'52.429"N, 21°14'39.718"E, 237 m a.s.l., site with dense bush and herbal cover and dominant herb *Solidagocanadensis*;

(**RC**) Cemetery, Rozália (Košice) – 48°43'56.473"N, 21°14'2.451"E; 248 m a.s.l., T_mean_: 10.3 ± 6.5 °C, site characterised by trees *Tiliacordata* and *Taxusbaccata* and herbal cover, highly managed site with intensive human intervention;

(**RL**) Fragment of a woodland, Račí potok (Košice) – 48°43'32.050"N, 21°14'0.144"E, 240 m a.s.l., T_mean_: 9.8 ± 5.0 °C, fragment of a thermophilous oak wood with *Tiliacordata*, *Cornusmas* and dense cover of *Hederahelix*;

(**VL**) Fragment of a woodland, Výmoľ (Košice) – 48°43'59.546"N, 21°16'49.022"E, 247 m a.s.l., T_mean_: 10.3 ± 5.5 °C, fragment of a thermophilous oak wood with dense bush and sparse herbal vegetation e.g., *Poanemoralis* and *Violaodorata*;

(**IA**) Thermophilous cornel-oak wood (ass. *Corneto*–*Quercetum*) in front of the cave entrance of Ardovská cave (Slovak Karst) – 48°31'16"N, 20°25'14"E, 317 m a.s.l., T_mean_: 9.6 ± 5.6 °C, scree slope with mosses and lacking a herbal cover, SW exposition (20° slope), distance to cave entrance ~ 45 m;

(**IS**) Thermophilous hornbeam wood with sparse *Acer* sp. and *Quercus* sp., (ass. *Waldsteinio*-*Carpinetum*) in front of the cave entrance of Silická ľadnica Ice Cave (Slovak Karst) – 48°32'55"N, 20°30'12"E, 480 m a.s.l., T_mean_: 8.5 ± 4.8 °C, scree slope with sparse herbal cover, W exposition (35° slope), distance to cave entrance ~77 m;

(**IZ**) Fragment of maple–hornbeam wood (ass. *Aceri*–*Carpinetum*) at the bottom of Zádiel Gorge (Slovak Karst) – 48°37'20"N, 20°54'54"E, 340 m a.s.l., T_mean_: 8.5 ± 4.6 °C, site near the bank of the Blatnický Creek, S exposition (5° slope);

(**IDS**) Coniferous wood (ass. *Fageto*-*Piceetum*) in front of the cave entrance of Dobšinská Ice Cave (Slovak Paradise) – 48°52'05"N, 20°18'14"E, 969 m a.s.l. (elevation of cave entrance), T_mean_: 4.6 ± 2.8 °C, site on the scree slope with herbal cover, N exposition (30° slope), distance to cave entrance ~ 17.3 m.

The maximum geographic distance between the urban and natural site (AT– IDS) was ~ 73 km. Geographic distances between urban sites were smaller than between natural sites (0.7–8.0 km and 7.0–53.0 km, respectively). Within urban sites, the minimum distance was ~ 0.7 km (between sites BC and BP), and within natural sites the minimum distance was ~ 7 km (between sites IA and IS).

Regarding soil sampling, the research adhered to the conditions of License #2661/2017-6.3. from the Ministry of the Environment of the Slovak Republic. The Collembola specimens were extracted from soil samples using a modified high-gradient apparatus (Crossley and Blair 1991). The samples were collected at study sites using soil corers 10 cm in diameter to a maximum depth of 8/10 cm. During extraction the specimens were fixed in pure ethyl alcohol. A total of 70 specimens from nine urban and four natural populations were used for molecular analyses (2–11 specimens from each population) (Table 1).

For the molecular study, the specimens were stored in ethanol (95.6%) at a temperature of -20 °C. DNA was extracted from the whole body of the collembolan specimens. Basic morphological observations were made using a Leica EZ4 D stereomicroscope. Since our effort required destructive DNA extraction, over 50% of the specimens analysed were then mounted on permanent microscopic slides for the taxonomic verification, with the numbers of specimens from the individual sites as follows: AR – 4, AT– 8, BC–10, BP – 8, NP – 5, PR – 7, RC – 6, RL– 6, IA – 9, IS – 9, IZ – 5 and IDS – 9 specimens. This procedure was carried out in a phase-contrast light microscope Carl Zeiss Axiolab A1 (Carl Zeiss Microscopy, Oberkochen, Germany). Molecular analyses were carried out at the Institute of Biology and Ecology, Faculty of Science, P. J. Šafárik University (IBE FS PJSU), Košice, Slovakia and Institute of Entomology, BC AV ČR, České Budějovice, Czech Republic. The collection of *Isotomiellaminor* specimens from the study sites is available at the IBE FS PJSU.

### ﻿DNA extraction, amplification, and sequencing

All DNA laboratory work was carried out using sterile barrier tips to prevent contamination. Using the DNeasy Blood and Tissue Kit from QIAGEN (Valencia, CA, USA), total genomic DNA was extracted from individuals using the following changes, per the manufacturer’s protocol: specimens were air-dried to remove excessive ethanol before DNA extraction. The individuals were then placed into sterile 1.5 ml Eppendorf tubes with 200 μl of lysis buffer (including Proteinase K) and incubated at 56 °C for about an hour and 40 minutes. Final elution was performed with 50 μl and stored at –20 °C.

To determine the genetic diversity among *Isotomiellaminor* populations, we attempted to use four molecular markers with different mutation rates that were commonly used in previous molecular genetic studies of springtails (von Saltzwedel et al. 2016, 2017; Zhang et al. 2018c; Lukić et al. 2019), i.e. the mitochondrial COI gene and 16S rDNA, as well as the nuclear 18S rDNA and 28S rDNA (D1-D2 region). Specifically, we used the following primers: LCO1490 (5’-GGTCAACAAATCATAAAGATATTGG-3’) and HCO2198 (5’-TAAACTTCAGGGTGACCAAAAAATCA-3’) for the COI gene (Folmer et al. 1994); N1-J-12585b (5’-CCCTTACGAATTTGAATATATCC-3’) and Coll_LRN-13486 (5’-CCGTGCWAA GGTAGCATAAT-3’) for the 16S rDNA (Simon et al. 1994); 28S rD1.2a (5’-CCCSSGTAATTTAAGCATATTA-3’) and 28SboutR (5’-CCCACAGCGCCAGTTCTGCTTACC-3’) for the 28S rDNA (Whiting 2002) and 18SF (5’-GTTCGATTCCGGAGAGGGA-3’) and 18SbiR (5’-GAG TCTCGTTCGTTATCGGA-3’) for the 18S rDNA (Whiting et al. 1997).

PCRs of these molecular markers were carried out using the Unis Taq system (TopBio s.r.o., Prague, Czech Republic). Reaction volumes (12.5 μl) consisted of 7.625 μl of ddH2O, 1 μl of template DNA (not quantified), 1.25 μl of 10× reaction buffer, 1 μl of dNTP mixture containing 2.5 mM of each dNTP, 0.75 μl of 5 μM solution of each primer and 0.125 μl of 0.5 U UNIS Taq polymerase. The PCRs were carried out in a thermocycler TProfessional TRIO Thermocycler (Biometra), and PCR conditions included one initial activation step at 94 °C for 2 min, followed by 35 amplification cycles of denaturation at 94 °C for 30 s, annealing at 43–45 °C (COI) or 45 °C (16S) or 55 °C (28S, 18S) for 30 to 45s depending on the marker, elongation at 72 °C for 1 min and a final elongation step at 72 °C for 2 min. After verification on agarose electrophoresis, the PCR products were cleaned enzymatically with ExoI-FastAP (Thermo Fisher Scientific) before direct sequencing by the Eurofins Genomic company (Ebersberg, Germany) using the Sanger method.

Chromatograms were checked and manually edited using Chromas v. 2.6.6 (Technelysium Pty Ltd, http://technelysium.com.au/wp/). All the sequences were also verified as collembolan DNA using the GenBank BLASTn search (the MegaBlast algorithm with the default setting brought alignments only to other collembolans with an E value < 4 × 10^-115^). Since the ORF of the COI sequences was correct and none of them had stop codons or indels, they were all assumed to be true mitochondrial and not nuclear copies (NUMTs) or pseudogenes.

The sequences are publicly available in GenBank. The GenBank accession number and voucher code for each specimen are listed in Suppl. material 1: table S1.

### ﻿Molecular data analyses

All methods and datasets used in the molecular analyses that are presented in the paragraphs below are summarised in Table 2. The DNA sequences for each marker were aligned separately using the ClustalW method (Thompson et al. 1994) in the MEGA X program (Kumar et al. 2018).

**Table 2.** Analytical steps, methodology, software, and data type for molecular analyses.

Analytical step	Method and software	Type of data
Variability of markers	MEGA X, DNAsp	COI, 28S rDNA, concatenated matrix
Computation of genetic distances	p-distance, K2P, MEGA X	96 COI dataset*, 28S rDNA dataset, Lineages of COI dataset
Phylogenetic analysis	Maximum Likelihood method	COI, 28S rDNA, concatenated matrix
Species delimitation	Assemble Species by Automatic Partitioning (ASAP), Poisson Tree Processes (bPTP)	96 COI dataset
Haplotype network	Minimum spanning network, PopArt	COI, 28S rDNA
Analysis of Molecular Variance (AMOVA)	ARLEQUIN v3.5	COI, 28S rDNA, concatenated matrix

* The 96 COI dataset involved populations of *I.minor* from urban-natural system, populations from von Saltzwedel et al. (2016) and *F.candida*, *F.fimetaria*, and *F.penicula* populations. Lineages of COI dataset generated by ASAP method involved *I.minor* populations from urban-natural system and from the study of von Saltzwedel et al. (2016).

In this study, we use the term lineage to refer to a clade at the highest level, which is not always morphologically distinct but is genetically determined (von Saltzwedel et al. 2016; Striuchkova et al. 2024). We used analysis on COI to designate MOTUs and on 28S data for qualitative confirmation of the COI results. We first delimited lineages (MOTUs - molecular operational taxonomic units) using the DNA barcoding region from 96 COI sequences. The dataset included our own sequences of *Isotomiellaminor* from urban-natural system (subfamily Anurophorinae, 70 sequences; see Suppl. material 1: table S1) as well as additional data from the GenBank and BOLD databases, comprising 13 *I.minor* sequences from nearby geographic regions (4 (HR1–4) from Croatia: KF684651-4; 4 (PL1–4) from Poland: KF684683-6; 5 (AT1Im1–5) from Austria: KF684646-50, GenBank, according to von Saltzwedel et al. (2016). Since in databases are no data available from the same *Isotomiella* genus, species of closely related genus “*Folsomia*” from the same subfamily were also included: *Folsomiacandida* Willem, 1902 - 5 sequences, GBCO3026-19, GBCO3284-19, BASS050-15, ECTO019-09 (BOLD), *Folsomiafimetaria* (Linnaeus, 1758) - 3 sequences, DKINV034-21, ECTTO023-09, ECTTO024-09 (BOLD), and *Folsomiapenicula* Bangal, 1939 - 5 sequences, COLLH3521-11, COLLH3522-11, GBCO3285-19, GBCO3286-19, GBMIN136228-18 (BOLD). To clarify possible MOTUs, two single locus lineage delimitation methods were applied. The distance-based method, Assemble Species by Automatic Partitioning (ASAP) (Puillandre et al. 2021) was performed on the ASAP online platform (https://bioinfo.mnhn.fr/abi/public/asap/asapweb.html) using two distance metrics: Kimura 2-parametr model (K2p, Kimura 1980) and uncorrected p-distances (p-dist) with default settings. For the tree-based Poisson Tree Processes (bPTP) model (Zhang et al. 2013), we reduced the dataset to unique haplotypes and create an unrooted maximum likelihood (ML) phylogenetic tree using IQ-TREE web server (http://iqtree.cibiv.univie.ac.at/; Trifinopoulos et al. 2016). Lineage delimitation was then conducted on the bPTP web server (http://species.h-its.org/ptp/) with default parameters.

Genetic distance estimates were used to assess the genetic variability of *I.minor* both within and among populations, as well as across different lineages. We used the MEGA X software (Nei and Kumar 2000) to calculate two distances, uncorrected p-dist and K2p, to ensure comparability our data with other similar studies carried out in Collembola. The extended dataset (96 COI sequences) was grouped by locality/site and by lineage (MOTU) as identified in the previous analysis. Analysis of 28S rDNA genetic distances included only urban-natural dataset since no data were available in databases for other outgroup species and populations of *I.minor* (von Saltzwedel et al. 2016).

To estimate phylogenetic relationships among *I.minor* populations and determine whether distinct lineages corresponded to specific collection sites, we generated phylograms using the Maximum Likelihood (ML)method. Phylograms were reconstructed from both single-locus and concatenated datasets using IQ-TREE web server, with models HKY+F+I+G4 (COI), HKY+F+G4 (28S) and TIM2+F+G4 (concatenated dataset) selected by the module Model Selection (Kalyaanamoorthy et al. 2017) and node support assessed by bootstrapping (1000 replicates). Relevant sequences of *Folsomiacandida* Willem, 1902 (subfamily Anurophorinae) (HQ732049 for COI and JN981046 for 28S rDNA, GenBank) were used as an outgroup.

Haplotype diversity (*H_d_*) was assessed using the DNAsp program (Rozas et al. 2017), which also generated haplotype files for lineage delimitation (bPTP) and subsequent analyses. Genetic structuring and potential barriers to dispersal were evaluated using ARLEQUIN v. 3.5 (Excoffier and Lischer 2010). An analysis of molecular variance (AMOVA) was performed for all markers (COI, 28S, and concatenated datasets), with haplotypes grouped according to habitat type (urban or natural). Standard AMOVA parameters were applied, including pairwise distances and 1000 permutations.

To visualise the relationships among haplotypes and their distribution at individual sites, we create haplotype networks (for COI and 28S) using the minimum spanning network (MSN) model (default epsilon = 0) in the PopArt program (Leigh and Bryant 2015).

## ﻿Results

Four markers were amplified; however, only the mitochondrial COI fragment (5´ part) and part of the 28S rDNA (D1-D2) locus yielded quality DNA sequences of *Isotomiellaminor*. For molecular analyses, 70 individuals were used (50 from urban and 20 from natural sites). Consequently, three datasets – COI, 28S and concatenated matrix (COI+28S) – were included in the further analyses. The final length of the alignments was 497 bp (COI), 744 bp (28S) and 1247 bp (concatenated sequences). Similar and generally high values of haplotype diversity were observed in the studied markers, COI: *H_d_* = 0.913; 28S: *H_d_* = 0.829; concatenated matrix: *H_d_* = 0.919.

**Figure 2.**
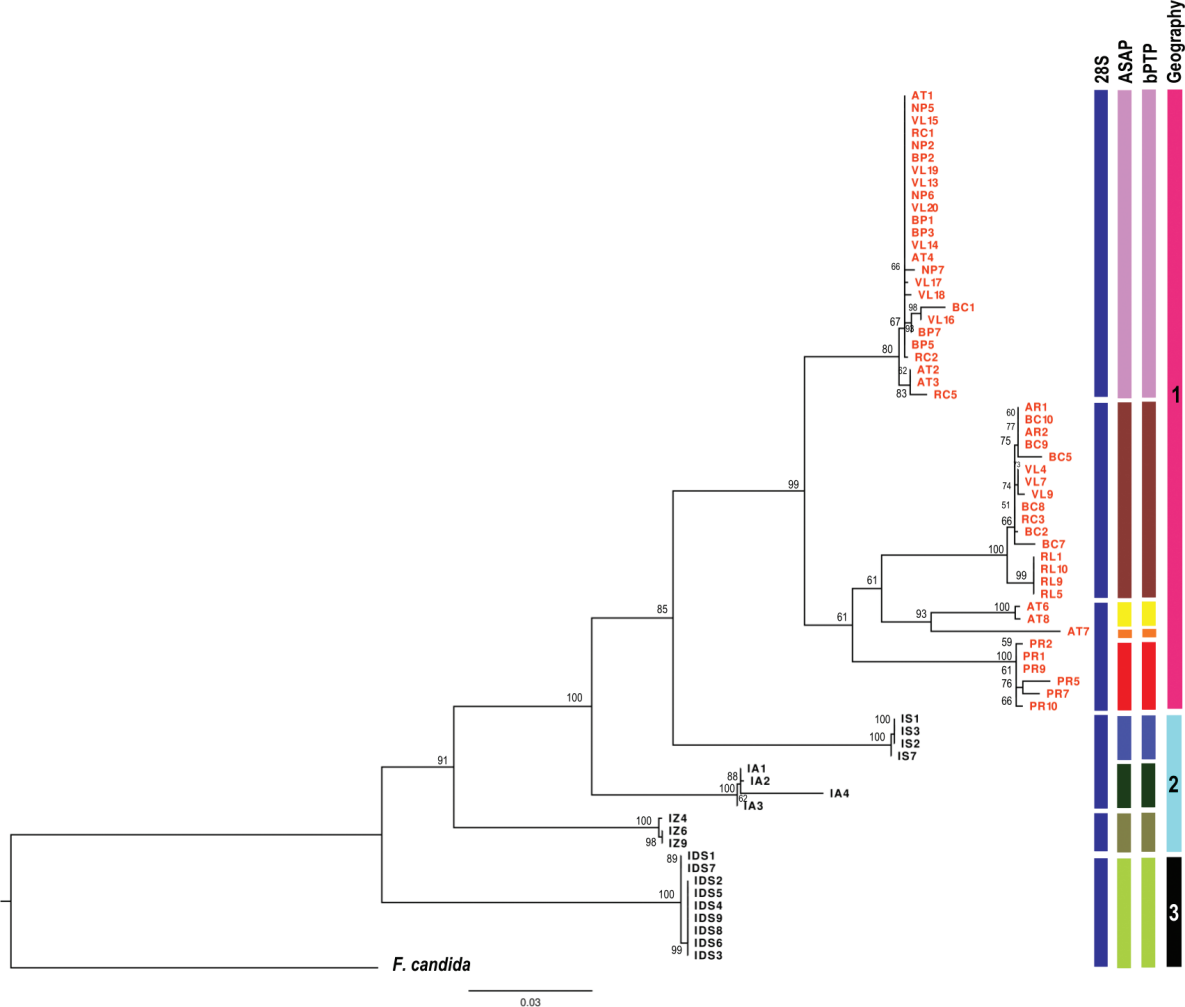
Maximum Likelihood tree of *Isotomiellaminor* populations inferred from the phylogenetic analysis of the concatenated dataset (ML, TIM2+F+G4, 1000× bootstrap; values > 50% shown) with *Folsomiacandida* as the outgroup (HQ732049 + JN981046). Blue columns designate 28S-based clusters, the delineations of MOTUs based on ASAP and bPTP analyses of COI indicated by different colours in columns. Geographic distributions of populations: 1 – Košice city, 2 – Slovak Karst, 3 – Slovak Paradise. For abbreviations of sampling sites, see the Materials and methods section, red symbols – urban sites, black symbols – natural sites.

The summary of the ASAP and bPTP delineation is provided in the Suppl. material 1: table S2, fig. S1A, B and is visualised in Fig. 2. Both distance metrics used in the ASAP method resulted in the same partitioning of subsets (Suppl. material 1: table S2), with slight differences in threshold distances: 8.56% for K2p and 8.15% for p-dist and histograms showing wide “barcoding gap” (Suppl. material 1: fig. S1A). Each outgroup species i.e. *F.fimetaria* and *F.penicula* (with exception of *F.candida*) formed a distinct group, while *I.minor* was divided into 10 lineages. Natural populations tended to cluster according to the given site, except for one subset that included individuals from Slovakia (IDS), Austria (AT1Im), and Poland (PLIm). Urban populations exhibited a different pattern; while some subsets were specific to single populations (PR, AT), most of the other urban populations (AT, BC, BP, NP, RC, RL, VL) were grouped into just two-three subsets.

Although the bPTP method is based on haplotypes, results were congruent with the ASAP partitioning, with high support (> 0.79, Suppl. material 1: fig. S1B). Exceptions are the Croatian *I.minor* samples (HR) and the outgroup species *F.fimetaria* and *F.penicula*, each of which split into two subgroups.

The genetic distances for the COI marker are presented in Table 3. Intrapopulation distances of *I.minor* ranged from 0.0 to 7.54% and from 0.0 to 8.30% (for p-dist and K2p, respectively). Intraspecific variability of outgroups was similar, ranging from 3.22 to 11.15% and from 3.37 to 12.78% (p-dist and K2p, respectively). Interpopulation distances ranged from 0.09 to 23.24% (p-dist) and 27.95% (K2p), while interspecific distances from 17.38% (p-dist) or 19.90% (K2p) to 22.78% (p-dist) or 27.41% (K2p).

**Table 3.** Genetic distances (%) of *Isotomiellaminor* within and between populations and outgroup species (*F.candida*, *F.fimetaria* and *F.penicula*) for COI marker.

Populations	Within populations/species (%)		Between populations/species (%)																		
	p-dist	K2P	AR	AT	BC	BP	NP	PR	RC	RL	VL	IA	IDS	IS	IZ	AT1Im	HRIm	PLIm	*F.candida*	*F.fimetaria*	*F.penicula*
**AR**	0.00	0.00	–	12.68	2.39	12.77	12.70	14.09	9.87	2.01	9.41	16.60	20.63	17.66	18.18	20.72	18.91	20.72	20.08	19.52	21.37
**AT**	7.54	8.30	14.19	–	12.00	5.06	5.12	13.76	7.17	13.39	7.18	16.34	19.88	16.51	19.64	19.89	19.75	20.01	21.05	19.58	22.43
**BC**	4.61	5.12	2.65	13.42	–	11.35	11.37	14.37	9.40	4.20	9.01	16.95	20.80	17.72	18.88	20.88	19.47	20.88	20.61	19.88	21.77
**BP**	0.14	0.14	14.28	5.55	12.67	–	0.23	13.08	3.76	13.37	3.62	16.09	19.41	15.54	20.08	19.41	19.01	19.41	21.36	19.21	22.39
**NP**	0.30	0.31	14.20	5.61	12.69	0.23	–	13.12	3.85	13.31	3.67	16.13	19.46	15.58	20.18	19.46	18.90	19.46	21.43	19.24	22.39
**PR**	0.75	0.76	15.87	15.51	16.22	14.66	14.70	–	13.71	14.26	13.49	14.09	19.51	18.58	18.79	19.60	17.90	19.20	19.57	17.38	19.77
**RC**	7.32	8.15	11.09	7.93	10.53	4.14	4.23	15.45	–	10.82	5.50	16.31	19.81	16.26	19.70	19.83	18.97	19.93	21.14	19.59	22.48
**RL**	0.00	0.00	2.05	15.13	4.53	15.07	14.98	16.09	12.20	–	10.40	17.15	22.04	17.66	18.38	22.13	19.52	22.13	20.52	19.32	21.33
**VL**	5.73	6.38	10.52	7.95	10.05	4.03	4.07	15.16	6.11	11.66	–	16.30	19.75	16.15	19.67	19.78	19.07	19.78	21.02	19.46	22.25
**IA**	0.20	0.20	18.92	18.64	19.38	18.30	18.35	15.68	18.57	19.67	18.56	–	17.26	17.05	16.36	17.35	18.86	17.76	19.22	18.23	20.23
**IDS**	0.16	0.16	24.25	23.26	24.49	22.60	22.66	22.65	23.14	26.32	23.05	19.69	–	20.32	20.30	0.09	22.59	1.10	21.01	20.84	22.73
**IS**	0.10	0.10	20.35	18.90	20.44	17.59	17.63	21.78	18.53	20.35	18.38	19.83	23.95	–	20.59	20.32	19.97	21.13	21.64	21.61	22.61
**IZ**	0.13	0.13	20.97	23.16	21.96	23.84	23.97	21.89	23.25	21.25	23.20	18.65	23.86	24.55	–	20.39	20.29	20.79	19.99	21.19	21.40
**AT1Im**	0.00	0.00	24.38	23.28	24.60	22.60	22.66	22.77	23.17	26.45	23.09	19.81	0.09	23.95	23.99	–	22.64	1.01	21.09	20.93	22.78
**HRIm**	1.74	1.79	21.88	23.13	22.65	22.05	21.90	20.54	21.99	22.72	22.12	21.84	26.99	23.40	23.83	27.06	–	23.24	20.80	21.09	21.13
**PLIm**	0.00	0.00	24.36	23.42	24.58	22.59	22.65	22.21	23.30	26.43	23.07	20.34	1.11	25.11	24.55	1.01	27.95	–	21.41	20.79	22.78
***F.candida***	11.15	12.78	23.57	24.90	24.30	25.33	25.43	22.76	25.03	24.22	24.86	22.32	24.82	25.91	23.41	24.92	24.54	25.37	–	18.90	19.76
***F.fimetaria***	3.76	3.94	22.82	22.98	23.34	22.46	22.49	19.90	22.99	22.54	22.79	21.04	24.66	25.98	25.25	24.79	24.99	24.57	21.96	–	19.75
***F.penicula***	3.22	3.37	25.32	26.94	25.91	26.89	26.90	23.10	27.02	25.26	26.66	23.79	27.34	27.09	25.40	27.41	25.01	27.39	23.23	23.25	–

Values below the diagonal indicate K2p-distance (K2p), above the diagonal p-distance (p-dist). Urban-natural dataset (red- urban populations) and populations of *I.minor* from the study of Saltzwedel et al. (2016): Austria (AT1Im), Croatia (HRIm) and Poland (PLIm). (for abbreviations of sites, see the Materials and methods section).

Genetic distances of *I.minor* within lineages (Table 4) were smaller than those within sampling sites (see above), with a minimum of 0.1% for both distance models and a maximum of 1.74% (p-dist) or 1.79% (K2p). In contrast, genetic distances between lineages were significantly larger, ranging from 10.87% (p-dist) or 11.98% (K2p) to 22.75% (p-dist) or 27.22% (K2p).

**Table 4.** Genetic distances (%) of *Isotomiellaminor* within and between lineages designated by MOTU analyses for COI marker.

Lineage	Intralineage (%)		Interlineage (%)									
	p-dist	K2p	L1	L2	L3	L4	L5	L6	L7	L8	L9	L10
**L1**	1.27	1.29	–	13.06	12.13	14.26	14.34	16.96	21.13	17.74	18.45	19.23
**L2**	0.64	0.64	14.66	–	10.87	11.61	13.23	16.07	19.42	15.61	20.11	19.01
**L3**	0.60	0.61	13.53	11.98	–	11.07	14.33	16.80	21.13	17.45	19.18	21.03
**L4**	–	–	16.24	12.86	12.27	–	14.53	17.61	20.88	19.27	19.58	21.73
**L5**	0.75	0.76	16.17	14.85	16.18	16.44	–	14.09	19.46	18.58	18.79	17.90
**L6**	0.20	0.20	19.39	18.25	19.24	20.38	15.68	–	17.40	17.05	16.36	18.86
**L7**	0.45	0.46	24.96	22.61	25.05	24.60	22.59	19.87	–	20.50	20.43	22.75
**L8**	0.10	0.10	20.45	17.68	20.12	22.68	21.78	19.83	24.21	–	20.59	19.97
**L9**	0.13	0.13	21.33	23.88	22.44	23.00	21.89	18.65	24.05	24.55	–	20.29
**L10**	1.74	1.79	22.31	22.05	25.99	25.99	20.54	21.84	27.22	23.83	23.83	–

Values below the diagonal indicate K2p-distance (K2p), above the diagonal, p-distance (p-dist). Lineages L1-L6 as in Fig. 2; L7(IDS1, IDS7, IDS2, IDS3, IDS4, IDS5, IDS6, IDS8, IDS9, AT1Im1, AT1Im2, AT1Im3, AT1Im4, AT1Im5, PLIm1, PLIm2, PLIm3, PLIm4); L8(IS1, IS2, IS3, IS7); L9(IZ4, IZ5, IZ9); L10(HRIm1, HRIm3, HRIm2, HRIm4), (for code abbreviations of sites, see the Materials and methods section and Suppl. material 1: table S2).

Regarding 28S marker, the genetic distances of *I.minor* from urban-natural system (Suppl. material 1: table S3) were smaller than those within COI marker. Intrapopulation distances ranged from 0.0 to 1.55% for p-dist and from 0.0 to 1.58% for K2p, respectively, while interpopulation distances from 0.0 to 3.88% for p-dist and from 0.0 to 4.00% for K2p.

The phylograms for the individual markers showed similar patterns despite the slight differences in topology (Suppl. material 1: fig. S2A, B). In the COI-based phylogram, the urban populations were divided into several smaller subclusters (independently of the given site), while four natural populations formed distinct (unique) subclusters. In contrast, in the 28S-based phylogram, the urban populations formed two larger subclusters, while the two natural populations, IA and IS, shared a single subcluster. The phylogram based on the concatenated dataset is complemented by the designated lineages (Fig. 2). The MOTUs, generated by both methods of species delimitation, corresponding to the topology of the ML tree. Both delimitation methods classified 70 *I.minor* specimens into nine MOTUs; natural specimens formed distinct (unique) groups, while urban specimens were intermixed. Despite a notable asymmetry in sampling—with fewer specimens from natural sites than from urban sites — the analyses remained consistent with respect to habitat/site. Regarding 28S as qualitative evidence for COIMOTUs, the pattern of high COI divergences in natural populations confirmed by 28S was relatively good supported, but high divergence in urban environment in COI was not always supported. Consequently, there are probably six supported MOTUs instead of nine. The urban populations were mixtures of different lineages and were clustered together, while the populations from natural sites formed separate groups, i.e., they were monophyletic and the genetic lineages were clearly grouped by site. This observed pattern was used as a hypothesis for the AMOVA analysis. Molecular variance was lowest within populations, while the highest values were found between groups: 56.19% for COI, 76.16% for 28S and 59.95% for the concatenated fragment (Table 5). The significantly high *F_ST_* values (0.862 for COI, 0.878 for 28S and 0.865 for the combined COI+28S) indicated strong group segregation and limited dispersal, further supporting the hypothesis.

**Table 5.** Analysis of molecular variance (AMOVA) of *I.minor* populations in the urban-natural system.

Source of variation	d.f.	Sum of squares				Variance components			Percentage of variance		
		COI	28S	COI+28S		COI	28S	COI+28S	COI	28S	COI+28S
Among groups	4	1081.487	278.495	1359.982	Va	24.426**	7.679***	32.105**	56.19	79.16	59.95
Among populations within groups	8	612.916	60.789	673.703	Vb	13.026***	1.175***	14.201***	29.97	11.66	26.52
Within populations	57	342.925	69.990	412.915	Vc	6.016***	1.228***	7.244***	13.84	12.18	13.53
Total	69	2037.329	409.271	2446.600		43.468	10.082	53.550			
Fixation Indices	COI: *FST* 0.862***				28S: *FST* 0.878***				COI+28S: *FST* 0.865***		

V – variance component, asterisks indicate significant differences * p < 0.05, ** p < 0.01, *** p < 0.001t, d.f. degrees of freedom.

**Figure 3.**
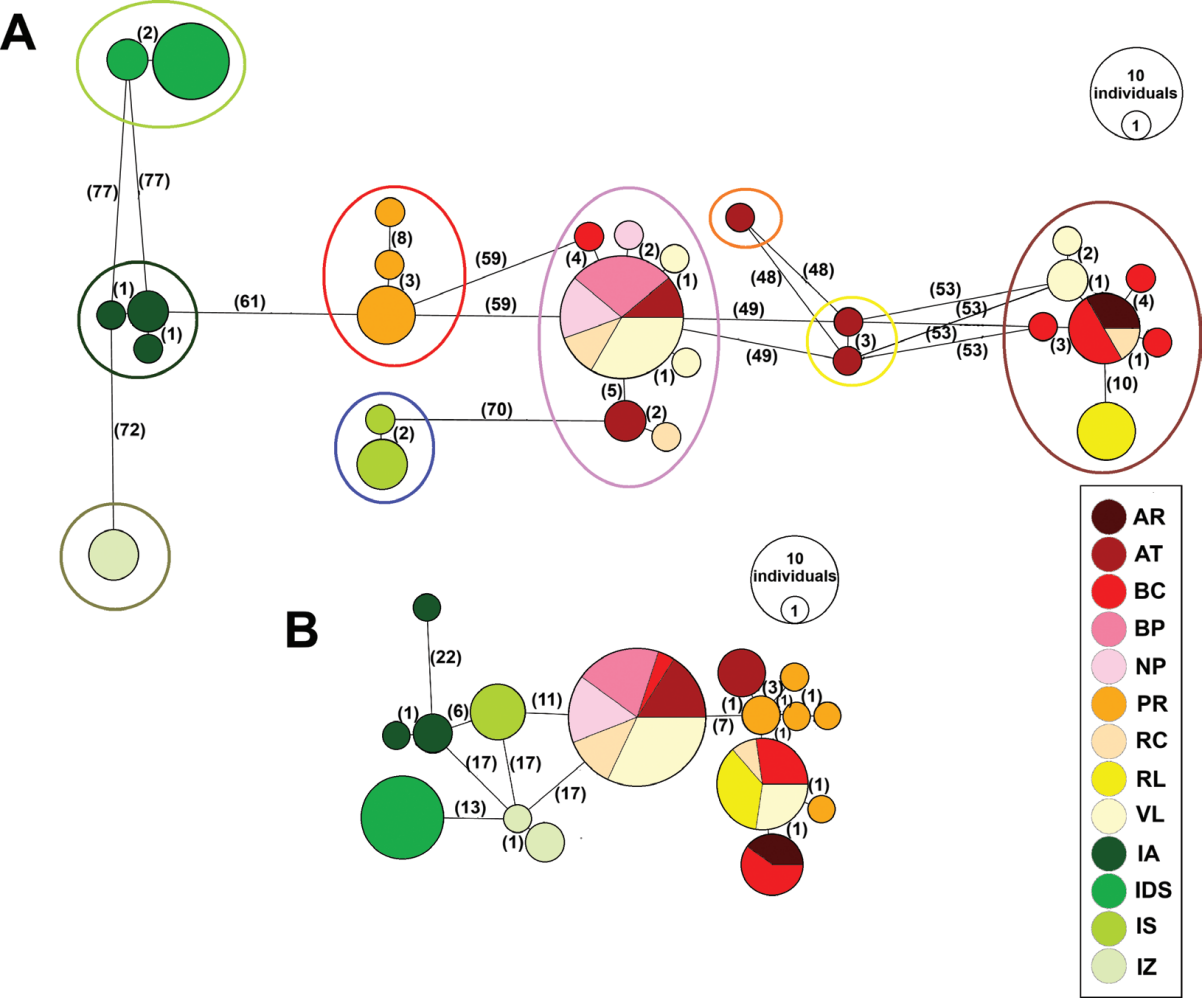
Minimum spanning haplotype networks showing the relationships among the haplotypes recorded in the 13 populations of *I.minor*. **A.**COI marker; **B.** 28S marker. The size of the circles is proportional to the number of individuals sharing the haplotype, and the line numbers reflect hypothetical mutational steps between the haplotypes, while lines without numbers represent a single mutational step. The colours represent the occurrence of the haplotypes in the populations studied (green – natural, yellow to red – urban). MOTUs/lineages are displayed by ellipses in colours corresponding to Fig. 2. For sampling site abbreviations, see the Materials and methods section and for the specimens haplotypes, see Suppl. material 1: table S1.

In addition to 70 sequenced individuals of *I.minor* for each marker, we recorded 28 haplotypes for COI and 16 for the 28S rDNA (Fig. 3A, B, Suppl. material 1: table S1). The most widespread COI haplotype was Hap2, found in the AT, BP, NP, RC, and VL populations, followed by Hap1, present in the AR, BC, and RC3 populations (Fig. 3A) Similarly, for 28S rDNA, Hp2 was the most widespread (AT, BC, BP, NP, RC, VL), with Hp4 occurring in the BC, RC, RL, and VL populations (Fig. 3B). Clear distinction between populations from urban and natural sites emerged, particularly in the 28S marker. None of the haplotypes were found in both types of sites (i.e., unique haplotypes for urban and unique for natural sites). Additionally, the number of mutations separating the COI haplotypes closely coincided with the delineated MOTUs from previous analyses.

## ﻿Discussion

In this study, a total of nine *Isotomiellaminor*MOTUs were generated by both species delimitation methods with a noticeable “barcoding gap”. A significant barcoding gap in the COI marker has been previously observed in closely related species of several collembolan genera (e.g., Sun et al. 2017; Zhang et al. 2018a, c). However, for reliable delimitation of species, further evidence is needed, such as observed differences in morphological and/or ecophysiological traits, geographical distributions, and also with independent (nuclear) markers. Although there was a correspondence between the MOTUs and their distributions in the three distinct geographical regions, the Slovak Karst (3 MOTUs), Slovak Paradise (1 MOTU) and Košice city (5 MOTUs), we did not notice any differences between these MOTUs in morphological traits. In addition, the independent genetic dataset with nuclear markers, which did not support some of the MOTUs, suggested that there was still some gene flow between them. Therefore, comparison of the COI and 28S datasets reveals only six species-level MOTUs, while the rest of the divergence found in the COI could then be considered as intraspecific/lineage/species-level MOTUs variability. Moreover, the MOTUs delineated on the COI gene corresponded with the tree topology and matched the following population structure. Individuals from natural populations grouped unequivocally by the natural site, whereas individuals from urban populations formed mixed clusters independent of the urban site origin.

In the present study, the same pattern of high variability between populations and low variability within populations was observed for both gene units (COI and 28S). The ASAP method is based on genetic distance analyses, with a threshold value being of importance. Hogg and Hebert (2004) assessed genetic variability of 19 Collembola species and set the threshold for delineation at 8% (p-dist), while Porco et al. (2014) in more extensive study adopted 14% threshold (K2p) to delineate MOTUs. Although studies on parthenogenetic springtails are limited, previous studies revealed high genetic variability forming a complex of cryptic lineages (MOTUs). For instance, in *Parisotomanotabilis* (Porco et al. 2012; von Saltzwedel et al. 2017; Striuchkova et al. 2022, 2024), high interlineage genetic distances were observed based on COI marker (p-dist: 14.18−19.82%, K2p: 15.77−23.35%). Similarly, in *I.minor* several distinct lineages based on COI were confirmed on a pan-European scale, with p-distances among populations 11–17% (von Saltzwedel et al. 2016). This aligns with the findings of the present study, which shows significant genetic distances between *I.minor* lineages (p-dist: 10.87−22.75%, K2p: 11.98−27.22%) and were similar to the genetic distances between outgroup species (p-dist: 17.38−22.78%, K2p: 19.90−27.41%). For comparison, Sun et al. (2018) noticed COI distances (K2p) 16.35−24.55% for closely related species of Collembola.

The cryptic diversity observed in natural populations of *I.minor* most likely resulted from the larger geographic distances and longer time of their isolation. The earlier isolation of natural populations and the very limited dispersal of MOTUs (lineages) between natural and urban populations were supported by AMOVA. The significantly high Fst values are consistent with other studies focused on *I.minor* and parthenogenetic *Parisotomanotabilis* (von Saltzwedel et al. 2016, 2017). Phylogenetic analysis indicated that populations in natural habitats exhibited greater intra-population stability, supported by the higher number of mutations detected between haplotypes. Haplotype analysis further demonstrated clear genetic differentiation between urban and natural populations of *I.minor* (especially in 28S marker) on a regional scale. Urban populations exhibited a higher number of haplotypes, likely due to increased genetic mixing, where smaller geographic distances and stronger anthropogenic disturbance contributed to a lack of population structure. In contrast, natural populations showed strong differentiation, with unique haplotypes and minimal sharing among populations. However, we are aware of sampling asymmetry with fewer samples from natural sites compared to urban ones in our study and so it is impossible to imply the founder effect, the phenomenon emphasised in genetic assessment of *I.minor* populations at the broad geographical scale (von Saltzwedel et al. 2016).

In this study, natural populations may be inferred as well-defined geographical isolates, supported by the high interpopulation genetic variability observed. The climatically inverse sites of the cave entrances/collapse dolines and deep gorges represent significant heterogenous environments of the karst landscape with specific topographic, microclimatic and vegetation conditions (Raschmanová et al. 2008, 2018), where strong selection may promote cryptic diversity in putatively common, ubiquitous species (Raschmanová et al. 2017). Also, habitats changed by anthropogenic disruption (including urban habitats) represent heterogenous environments and may be inhabited by a complex of cryptic lineages in Collembola (Striuchkova et al. 2022). Urbanisation and anthropogenic activities create a variety of biotopes with different microclimatic conditions, thus driving cryptic speciation (Marcelino et al. 2016; Hending 2024).

As has been noted in previous studies (Zhang et al. 2018b; Striuchkova et al. 2022; Striuchkova and Kuznetsova 2024), environmental conditions may lead to lineage specialisation. In widespread species such as *L.lanuginosus* and *P.notabilis* different genetic lineages from contrasting habitats were found. The authors suggested that ecological divergence of lineages may be preliminarily assessed by their preference for certain habitats. In present study, urban and natural environments differed in microclimatic conditions. The maximum difference in soil mean temperature between the urban and natural site was ∼ 7.2 °C. Our previous study investigated physiological traits in some urban (BP, NP, VL) and natural (IA, IS, IDS) populations and showed that urban and natural habitats inhabited physiologically different populations in terms of cold tolerance. This is in congruence with findings that urban and natural environments are inhabited by distinct genetic lineages. An ecological filtering of *Isotomiellaminor*MOTUs could be suggested between urban and natural environments, driven by habitat preferences and their adaptation to specific environmental conditions. This phenomenon can contribute to the evolution of parthenogenetic species in terrestrial arthropods (von Saltzwedel et al. 2014). Regarding relationship between genetic lineages and ecophysiological responses, the COI sequences could provide some informative baseline for thermal adaptation in Collembola. Interesting correlations were provided by some studies, for instance Collins and Hogg (2016) investigated genetic variability in the barcoding COI region in an Antarctic springtail *Gomphiocephalushodgsoni* Carpenter, 1908, which also showed considerable physiological variation in cold tolerance among individuals. The COI sequences of this species were considered as a sensitive genetic indicator. Similarly, Carapelli et al. (2019) examined the mitogenomes of several springtails and found evidence of selection, suggesting that survival under extreme environmental conditions may be associated with mtDNA modifications. However, in the case of *I.minor* we had a limited ability to correlate observed genetic differences with physiological traits directly, since for molecular and physiological aspect of study not identical specimens were tested. Thus, suggested assumption that adaptation of genetic lineages to specific environmental conditions may be of ecophysiological nature remains still unclear, although it seems convincing at this point.

## ﻿Conclusions

This study is a first attempt to examine structure of genetic variation in parthenogenetic and ubiquitous collembolan *Isotomiellaminor* in contrasting landscapes, representing an urban–natural system. Its populations inhabiting heterogenous urban–natural environment represented genetically different lineages. Further investigations in *Isotomiella* populations are needed, with larger molecular datasets including additional mitochondrial and nuclear markers, such as highly variable microsatellites, which could potentially shed more light on the issues of the cryptic speciation of *I.minor* populations in the studied urban–natural system. Also, investigation of eco-physiological traits of genetic lineages is required, since ecological filtering and biotic interactions jointly shape these traits, and it could partly explain evolutionary processes in this widespread parthenogenetic species in contrasting environments.

## ﻿Additional information

### Author ORCIDs

Mária Fedičová https://orcid.org/0009-0000-2109-7567

Natália Raschmanová https://orcid.org/0000-0002-9476-1753

Martina Žurovcová https://orcid.org/0000-0003-1539-9774

Vladimír Šustr https://orcid.org/0000-0003-2983-1855

Ľubomír Kováč https://orcid.org/0000-0001-8194-2128

### Data availability

All of the data that support the findings of this study are available in the main text or Supplementary Information.

## Supplementary materials

Supplementary material 1 Additional information Data type docx Explanation note **table S1.** GenBank accession number of COI and 28S rDNA sequences; distribution specimens to the haplotypes (separately for COI and 28S sequences). **table S2.** Delimitation of *I.minor*MOTUs based on COI marker by ASAP (p-distance: threshold 8.15%, and K2p: threshold 8.56%) and bPTP. **fig. S1. A.**ASAP histograms of pairwise distances (p-distance and K2P distance) based on 96 COI dataset; **B.**bPTP – Maximum likelihood dendrogram of haplotypes based on 96 COI dataset of *I.minor* sequences. **table S3.** Genetic distances (%) of *Isotomiellaminor* within and between populations for 28S rDNA marker. **fig. S2.** Maximum likelihood tree of *Isotomiellaminor* populations. **A.**COI marker, *Folsomiacandida* (HQ732049) as the outgroup. **B.** 28S marker, *Folsomiacandida* (JN981046) as the outgroup. https://binary.pensoft.net/file/1373312This dataset is made available under the Open Database License (http://opendatacommons.org/licenses/odbl/1.0/). The Open Database License (ODbL) is a license agreement intended to allow users to freely share, modify, and use this Dataset while maintaining this same freedom for others, provided that the original source and author(s) are credited.Mária Fedičová, Natália Raschmanová, Martina Žurovcová, Vladimír Šustr, Ľubomír Kováč
