# Supplementary material for: ﻿Structure of the genetic variation in the common springtail Isotomiellaminor (Hexapoda, Collembola) from contrasting habitats: evidence for different genetic lineages at a regional scale?
Source: Zookeys. 2025 Jul 14;1245:19–39. doi: 10.3897/zookeys.1245.152112 (PMC12280966; doi:10.3897/zookeys.1245.152112)
Supplement: Supplementary material 1 — Additional information [file zookeys-1245-019_article-152112__-s001.docx]

**Supplementary material 1**

Title: **Structure of the genetic variation in the common springtail *Isotomiella minor* (Hexapoda, Collembola) from contrasting habitats: evidence for different genetic lineages at a regional scale?**

Authors: Mária Fedičová^1^, Natália Raschmanová^1^*, Martina Žurovcová^2^, Vladimír Šustr^3^, Ľubomír Kováč^1^

^1^Department of Zoology, Faculty of Science, Institute of Biology and Ecology, P. J. Šafarik University in Košice, Košice, Slovak Republic,

^2^Institute of Entomology, Biology Centre AS CR v. v. i., Česke Budějovice, Czech Republic,

^3^Institute of Soil Biology and Biogeochemistry, Biology Centre AS CR v. v. i., Česke Budějovice, Czech Republic

- Corresponding author: [natalia.raschmanova@upjs.sk](mailto:natalia.raschmanova@upjs.sk) (N. Raschmanová)

**Table S1.** GenBank accession number of COI and 28S rDNA sequences; distribution specimens to the haplotypes (separately for COI and 28S sequences), (for code abbreviations of specimens, see the Materials and methods).

| Specimens | GenBank accession number | |  | Haplotype number | Sequences belonging to haplotype  (COI) |  | Haplotype number | Sequences belonging to haplotype  (28S) |
| --- | --- | --- | --- | --- | --- | --- | --- | --- |
|  | COI | 28S |  |  |  |  |  |  |
| AR1 | PP301898 | PP296995 |  | Hap 1 | AR1 |  | Hp1 | AR1 |
| AR2 | PP301899 | PP296996 |  |  | AR2 |  |  | AR2 |
| AT1 | PP301900 | PP296997 |  |  | BC8 |  |  | BC5 |
| AT2 | PP301901 | PP296998 |  |  | BC9 |  |  | BC9 |
| AT3 | PP301902 | PP296999 |  |  | BC10 |  |  | BC10 |
| AT4 | PP301903 | PP297000 |  |  | RC3 |  | Hp2 | AT1 |
| AT6 | PP301904 | PP297001 |  | Hap 2 | AT1 |  |  | AT2 |
| AT7 | PP301905 | PP297002 |  |  | AT4 |  |  | AT3 |
| AT8 | PP301906 | PP297003 |  |  | BP1 |  |  | AT4 |
| BC1 | PP301907 | PP297004 |  |  | BP2 |  |  | BC1 |
| BC2 | PP301908 | PP297005 |  |  | BP3 |  |  | BP1 |
| BC5 | PP301909 | PP297006 |  |  | BP5 |  |  | BP2 |
| BC7 | PP301910 | PP297007 |  |  | BP7 |  |  | BP3 |
| BC8 | PP301911 | PP297008 |  |  | NP2 |  |  | BP5 |
| BC9 | PP301912 | PP297009 |  |  | NP5 |  |  | BP7 |
| BC10 | PP301913 | PP297010 |  |  | NP6 |  |  | NP2 |
| BP1 | PP301914 | PP297011 |  |  | RC1 |  |  | NP5 |
| BP2 | PP301915 | PP297012 |  |  | RC2 |  |  | NP6 |
| BP3 | PP301916 | PP297013 |  |  | VL13 |  |  | NP7 |
| BP5 | PP301917 | PP297014 |  |  | VL14 |  |  | RC1 |
| BP7 | PP301918 | PP297015 |  |  | VL15 |  |  | RC2 |
| NP2 | PP301919 | PP297016 |  |  | VL16 |  |  | RC5 |
| NP5 | PP301920 | PP297017 |  |  | VL19 |  |  | VL13 |
| NP6 | PP301921 | PP297018 |  |  | VL20 |  |  | VL14 |
| NP7 | PP301922 | PP297019 |  | Hap 3 | AT2 |  |  | VL15 |
| PR1 | PP301923 | PP297020 |  |  | AT3 |  |  | VL16 |
| PR2 | PP301924 | PP297021 |  | Hap 4 | AT6 |  |  | VL17 |
| PR5 | PP301925 | PP297022 |  | Hap 5 | AT7 |  |  | VL18 |
| PR7 | PP301926 | PP297023 |  | Hap 6 | AT8 |  |  | VL19 |
| PR9 | PP301927 | PP297024 |  | Hap 7 | BC1 |  |  | VL20 |
| PR10 | PP301928 | PP297025 |  | Hap 8 | BC2 |  | Hp3 | AT6 |
| RC1 | PP301929 | PP297026 |  | Hap 9 | BC5 |  |  | AT7 |
| RC2 | PP301930 | PP297027 |  | Hap 10 | BC7 |  |  | AT8 |
| RC3 | PP301931 | PP297028 |  | Hap 11 | NP7 |  | Hp4 | BC2 |
| RC5 | PP301932 | PP297029 |  | Hap 12 | PR1 |  |  | BC7 |
| RL1 | PP301933 | PP297030 |  |  | PR2 |  |  | BC8 |
| RL5 | PP301934 | PP297031 |  |  | PR9 |  |  | RC3 |
| RL9 | PP301935 | PP297032 |  |  | PR10 |  |  | RL1 |
| RL10 | PP301936 | PP297033 |  | Hap 13 | PR5 |  |  | RL5 |

| Specimens | GenBank accession number | |  | Haplotype number | Sequences belonging to haplotype  (COI) |  | Haplotype number | Sequences belonging to haplotype  (28S) |
| --- | --- | --- | --- | --- | --- | --- | --- | --- |
|  | COI | 28S |  |  |  |  |  |  |
| VL4 | PP301937 | PP297034 |  | Hap 14 | PR7 |  | Hp4 | RL9 |
| VL7 | PP301938 | PP297035 |  | Hap 15 | RC5 |  |  | RL10 |
| VL9 | PP301939 | PP297036 |  | Hap 16 | RL1 |  |  | VL4 |
| VL13 | PP301940 | PP297037 |  |  | RL5 |  |  | VL7 |
| VL14 | PP301941 | PP297038 |  |  | RL9 |  |  | VL9 |
| VL15 | PP301942 | PP297039 |  |  | RL10 |  | Hp5 | PR1 |
| VL16 | PP301943 | PP297040 |  | Hap 17 | VL4 |  |  | PR9 |
| VL17 | PP301944 | PP297041 |  |  | VL7 |  | Hp6 | PR2 |
| VL18 | PP301945 | PP297042 |  | Hap 18 | VL9 |  | Hp7 | PR5 |
| VL19 | PP301946 | PP297043 |  | Hap 19 | VL17 |  | Hp8 | PR7 |
| VL20 | PP301947 | PP297044 |  | Hap 20 | VL18 |  | Hp9 | PR10 |
| IA1 | PP301948 | PP297045 |  | Hap 21 | IA1 |  | Hp10 | IA1 |
| IA2 | PP301949 | PP297046 |  |  | IA2 |  |  | IA3 |
| IA3 | PP301950 | PP297047 |  | Hap 22 | IA3 |  | Hp11 | IA2 |
| IA4 | PP301951 | PP297048 |  | Hap 23 | IA4 |  | Hp12 | IA4 |
| IDS1 | PP301952 | PP297049 |  | Hap 24 | IDS1 |  | Hp13 | IDS1 |
| IDS2 | PP301953 | PP297050 |  |  | IDS7 |  |  | IDS2 |
| IDS3 | PP301954 | PP297051 |  | Hap 25 | IDS2 |  |  | IDS3 |
| IDS4 | PP301955 | PP297052 |  |  | IDS3 |  |  | IDS4 |
| IDS5 | PP301956 | PP297053 |  |  | IDS4 |  |  | IDS5 |
| IDS6 | PP301957 | PP297054 |  |  | IDS5 |  |  | IDS6 |
| IDS7 | PP301958 | PP297055 |  |  | IDS6 |  |  | IDS7 |
| IDS8 | PP301959 | PP297056 |  |  | IDS8 |  |  | IDS8 |
| IDS9 | PP301960 | PP297057 |  |  | IDS9 |  |  | IDS9 |
| IS1 | PP301961 | PP297058 |  | Hap 26 | IS1 |  | Hp14 | IS1 |
| IS2 | PP301962 | PP297059 |  |  | IS2 |  |  | IS2 |
| IS3 | PP301963 | PP297060 |  |  | IS3 |  |  | IS3 |
| IS7 | PP301964 | PP297061 |  | Hap 27 | IS7 |  |  | IS7 |
| IZ4 | PP301965 | PP297062 |  | Hap 28 | IZ4 |  | Hp15 | IZ4 |
| IZ6 | PP301966 | PP297063 |  |  | IZ6 |  | Hp16 | IZ6 |
| IZ9 | PP301967 | PP297064 |  |  | IZ9 |  |  | IZ9 |

**Table S1.** Cont.

**Table S2.** Delimitation of *I. minor* MOTUs based on COI marker by ASAP (p-distance: threshold 8.15%, and K2p: threshold 8.56%) and bPTP.

| ASAP subset | | bPTP subset | (COI) Sequences belonging to the group |
| --- | --- | --- | --- |
| p-dist | K2p |  |  |
| 1 | 1 | I | **AR1 AR2 BC8 BC9 BC10 RC3 VL4 VL7 BC2 VL9 BC7 BC5 RL1 RL5 RL9 RL10** |
| 2 | 2 | II | **AT1 AT4 BP1 BP2 BP3 BP5 NP2 NP5 NP6 RC1 VL13 VL14 VL15 VL19 VL20 RC2 VL17 BP7 VL18 VL16 NP7 AT2 AT3 RC5 BC1** |
| 3 | 3 | III | **AT6 AT8** |
| 4 | 4 | IV | **AT7** |
| 5 | 5 | V | **PR1 PR2 PR9 PR10 PR7 PR5** |
| 6 | 6 | VI | **IA1, IA2, IA3, IA4** |
| 7 | 7 | VII | **IDS1 IDS7 IDS2 IDS3 IDS4 IDS6 IDS5 IDS8 IDS9** AT1Im1 AT1Im2 AT1Im3 AT1Im4 AT1Im5 PLIm1 PLIm2 PLIm3 PLIm4 |
| 8 | 8 | VIII | **IS1 IS2 IS3 IS7** |
| 9 | 9 | IX | **IZ4, IZ6, IZ9** |
| 10 | 10 | X | HRIm1, HRIm3 |
|  |  | XI | HRIm2, HRIm4 |
| 11 | 11 | XII | Foca Canada, Foca Germany, Foca France |
| 12 | 12 | XIII | Foca Netherland, Foca Slovakia |
| 13 | 13 | XIV | Fope Slovakia1, Fope Slovakia2, Fope Slovakia3 |
|  |  | XV | Fope Moldova1, Fope Moldova2 |
| 14 | 14 | XVI | Fofi Denmark1 |
|  |  | XVII | Fofi Denmark2, Fofi Denmark3 |
| 96 COI dataset included specimens from urban-natural system of the present study (marked by bold), from the study of Saltzwedel et al. (2016), namely Austria (AT1Im1, AT1Im2, AT1Im3, AT1Im4, AT1Im5), Poland (PLIm1, PLIm2, PLIm3, PLIm4) and Croatia (HRIm1, HRIm2, HRIm3, HRIm4), and three species of the genus *Folsomia*: *F. candida* (Foca), *F. penicula* (Fope) and *F. fimetaria* (Fofi) (for abbreviations of sampling sites, see the Materials and methods section). | | | |


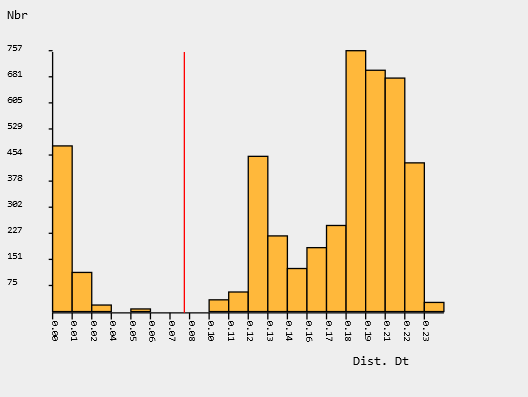

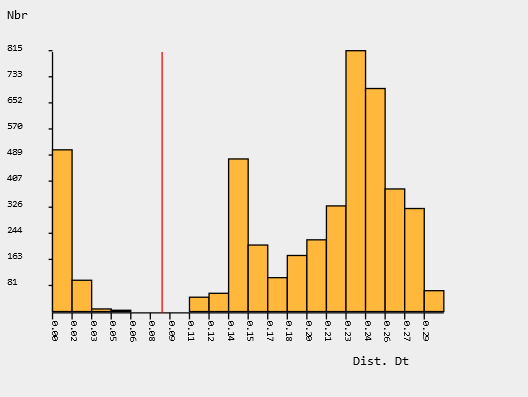


K2p

p - distance

**Figure S1. A** ASAP histograms of pairwise distances (p-distance and K2P distance) based on 96 COI dataset.


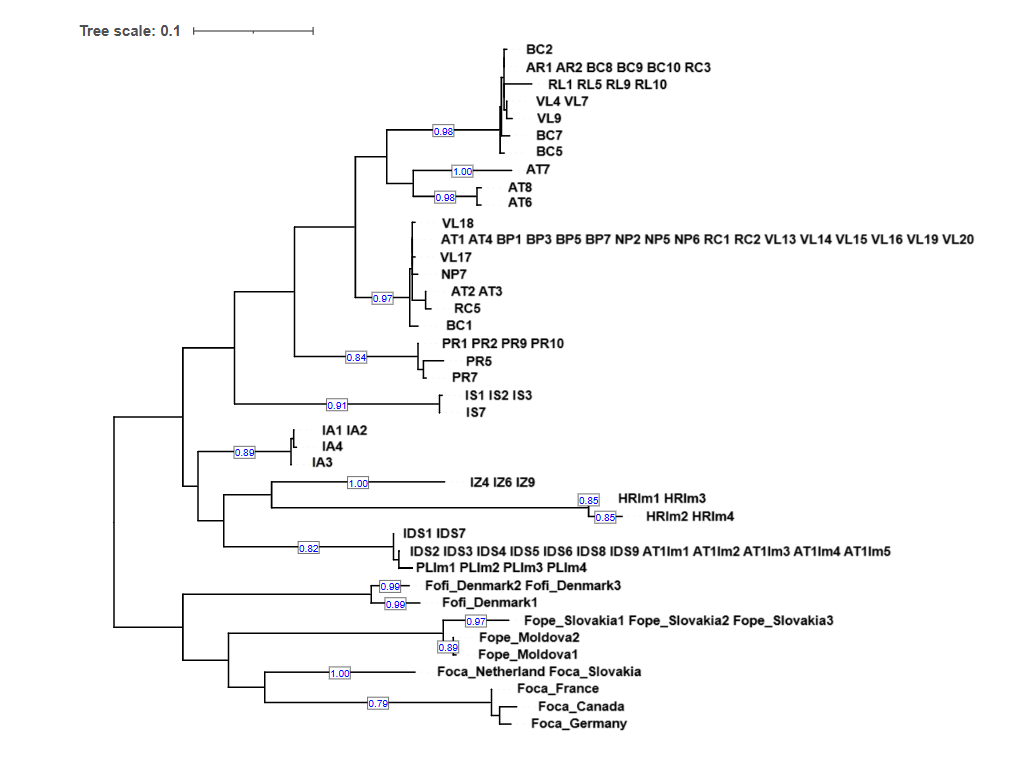


**Figure S1. B** bPTP – Maximum likelihood dendrogram of haplotypes based on 96 COI dataset of *I. minor* sequences (urban-natural system, for code abbreviations of specimens, see Suppl. Table S1), rooted by cluster of *Folsomia* sequences. Additional sequences of *I. minor* (AT1Im1–5; PL1–4; HR1–4, von Saltzwedel et al. 2016), *Folsomia candida* (Foca Canada, Foca Germany, Foca France), *Folsomia fimetaria* (Fofi Denmark1–3) and *Folsomia penicula* (Fope Moldova1–2, Fope Slovakia1–3).

| **Populations** | **Within populations (%)** | | **Between populations (%)** | | | | | | | | | | | | | | |
| --- | --- | --- | --- | --- | --- | --- | --- | --- | --- | --- | --- | --- | --- | --- | --- | --- | --- |
|  | **p-dist** | **K2P** | **AR** | **AT** | **BC** | **BP** | **NP** | **PR** | **RC** | **RL** | **VL** | **IA** | **IDS** | **IS** | **IZ** | |  |
| **AR** | 0.00 | 0.00 | ⎯ | 0.87 | 0.23 | 1.21 | 1.21 | 0.43 | 0.94 | 0.13 | 0.92 | 2.87 | 3.10 | 1.75 | 3.19 | |  |
| **AT** | 0.62 | 0.62 | 0.87 | ⎯ | 0.75 | 0.46 | 0.46 | 0.80 | 0.53 | 0.73 | 0.54 | 2.83 | 3.25 | 1.72 | 2.75 | |  |
| **BC** | 0.39 | 0.39 | 0.23 | 0.76 | ⎯ | 0.98 | 0.98 | 0.47 | 0.79 | 0.21 | 0.77 | 2.83 | 3.14 | 1.72 | 3.40 | |  |
| **BP** | 0.00 | 0.00 | 1.23 | 0.47 | 0.99 | ⎯ | 0.00 | 1.15 | 0.27 | 1.80 | 0.29 | 2.60 | 3.37 | 1.48 | 2.52 | |  |
| **NP** | 0.00 | 0.00 | 1.23 | 0.47 | 0.99 | 0.00 | ⎯ | 1.15 | 0.27 | 1.80 | 0.29 | 2.60 | 3.37 | 1.48 | 2.25 | |  |
| **PR** | 0.38 | 0.38 | 0.43 | 0.81 | 0.47 | 1.16 | 1.16 | ⎯ | 0.93 | 0.29 | 0.91 | 3.15 | 3.17 | 2.50 | 3.13 | |  |
| **RC** | 0.54 | 0.54 | 0.95 | 0.53 | 0.80 | 0.27 | 0.27 | 0.94 | ⎯ | 0.81 | 0.42 | 2.67 | 3.30 | 1.55 | 2.66 | |  |
| **RL** | 0.00 | 0.00 | 0.13 | 0.74 | 0.21 | 1.09 | 1.09 | 0.29 | 0.82 | ⎯ | 0.78 | 2.87 | 3.10 | 1.75 | 3.60 | |  |
| **VL** | 0.47 | 0.48 | 0.93 | 0.54 | 0.78 | 0.30 | 0.30 | 0.92 | 0.42 | 0.79 | ⎯ | 2.68 | 3.30 | 1.56 | 2.67 | |  |
| **IA** | 1.55 | 1.58 | 2.94 | 2.90 | 2.90 | 2.66 | 2.66 | 3.24 | 2.73 | 2.94 | 2.74 | ⎯ | 3.88 | 1.52 | 3.60 | |  |
| **IDS** | 0.00 | 0.00 | 3.17 | 3.33 | 3.21 | 3.45 | 3.45 | 3.24 | 3.38 | 3.17 | 3.38 | 4.00 | ⎯ | 3.64 | 1.84 | |  |
| **IS** | 0.00 | 0.00 | 1.78 | 1.74 | 1.74 | 1.50 | 1.50 | 2.08 | 1.57 | 1.78 | 1.58 | 1.54 | 3.74 | ⎯ | 2.52 | |  |
| **IZ** | 0.09 | 0.09 | 3.27 | 2.81 | 3.11 | 2.57 | 2.57 | 3.20 | 2.71 | 3.13 | 2.72 | 3.14 | 1.87 | 2.56 | ⎯ | |  |
| Values below the diagonal indicate K2p-distance (K2p), above the diagonal p-distance (p-dist). Urban-natural dataset (red – urban populations) (for abbreviations of sites, see the Materials and methods section). | | | | | | | | | | | | | | | |  |  |

**Table S3.** Genetic distances (%) of *Isotomiella minor* within and between populations for 28S rDNA marker.


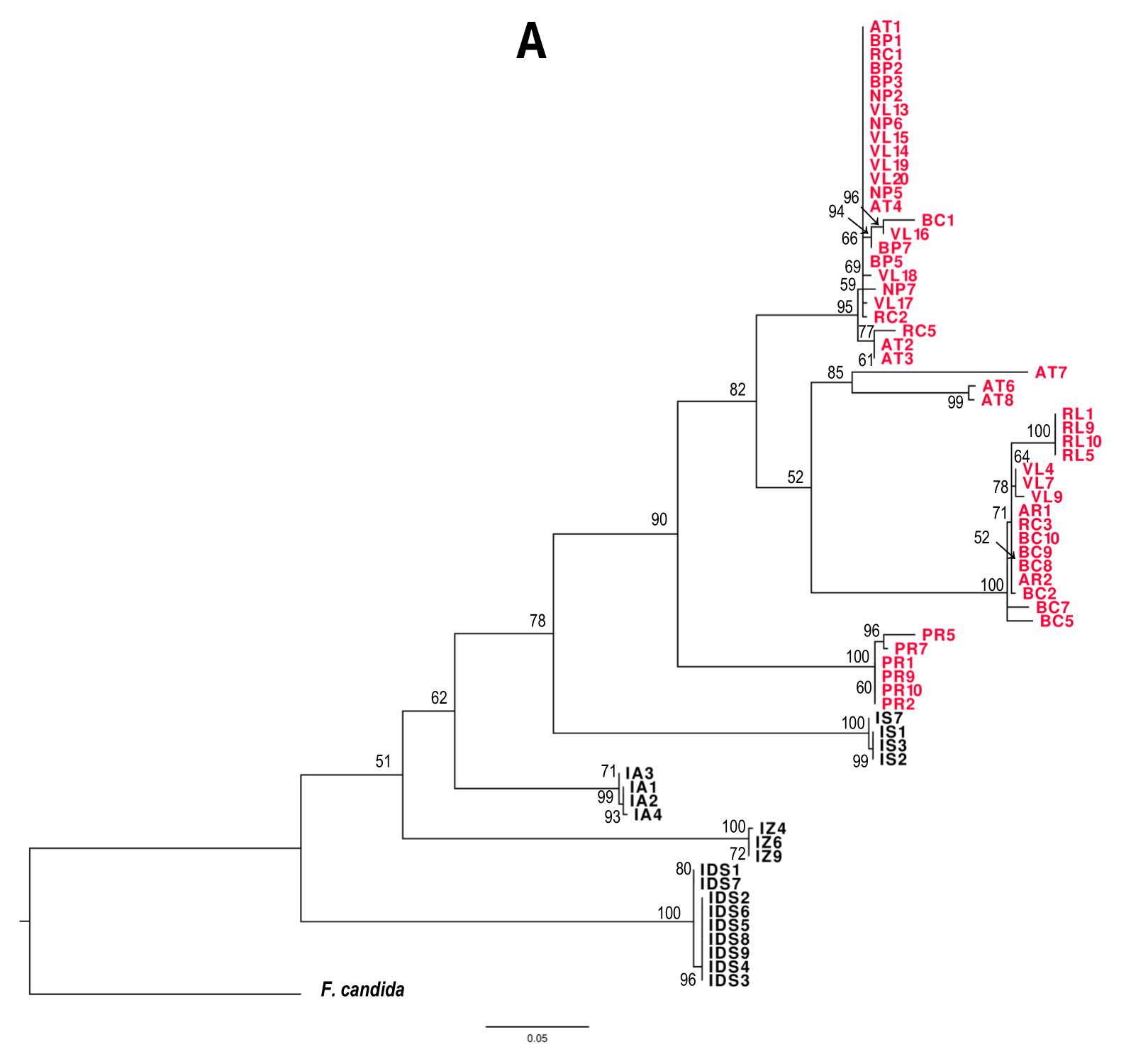


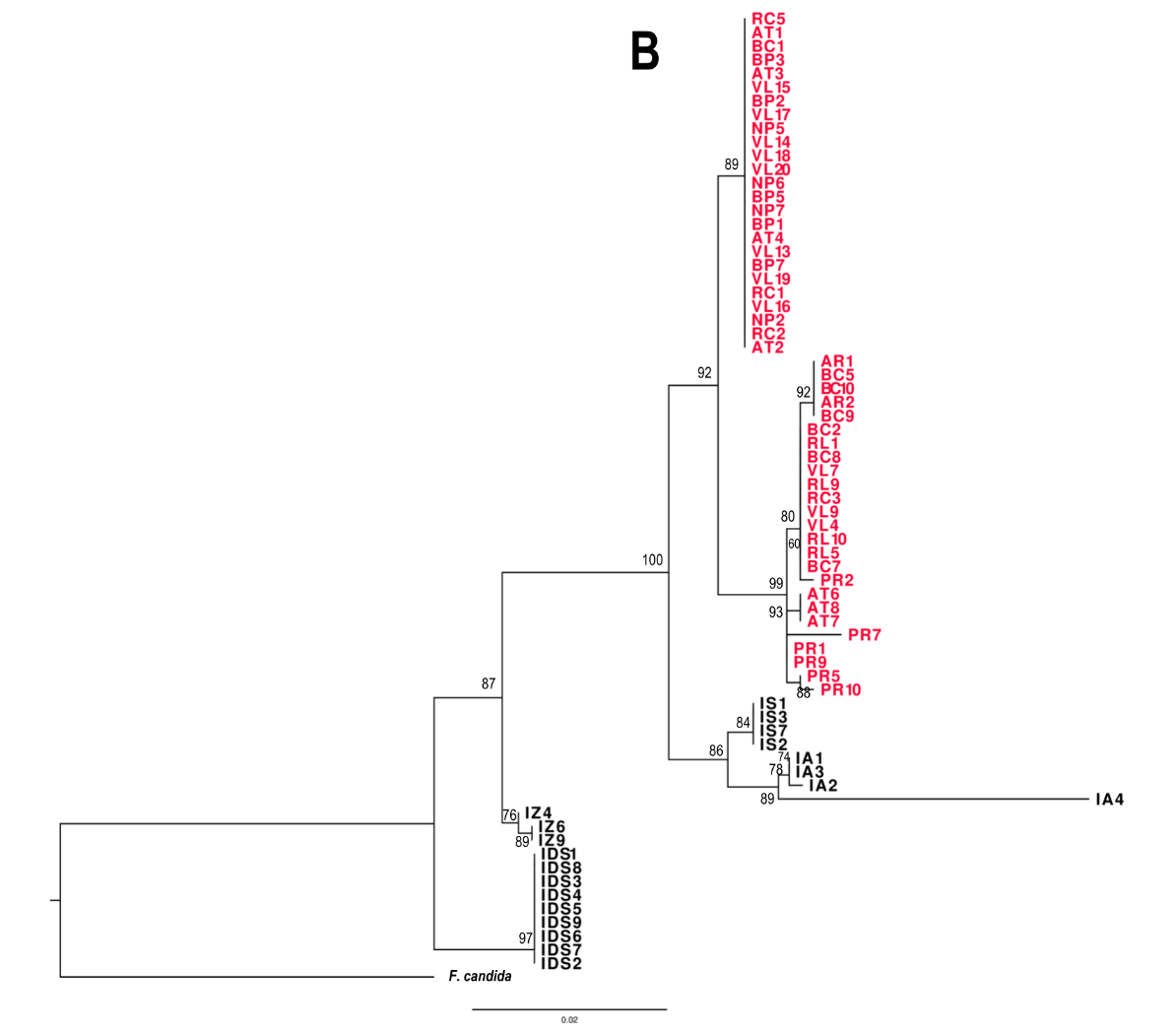


**Figure S2.** Maximum likelihood tree of *Isotomiella minor* populations. **A** COI marker, *Folsomia candida* (HQ732049) as the outgroup. **B** 28S marker, *Folsomia candida* (JN981046) as the outgroup. The models HKY+F+I+G4 (COI), HKY+F+G4 (28S) were used to construct the phylograms. Bootstrap values greater than 50% (1000 replicates) are displayed next to the branches (for abbreviations of sampling sites, see the Materials and methods section; red indicates urban specimens, black indicates natural specimens).
